# The information imperative: to study the impact of informational discontinuity on clinical decision making among doctors

**DOI:** 10.1186/s12911-020-01190-2

**Published:** 2020-07-28

**Authors:** Naveen R. Gowda, Atul Kumar, Sanjay K. Arya, Vikas H

**Affiliations:** 1 Department of Hospital Administration, All India Institute of Medical Sciences (AIIMS), New Delhi, India; 2 ESIC Hospital & Dental College, New Delhi, 110085 India

**Keywords:** Continuity of care, Information discontinuity, Clinical decision support

## Abstract

**Background:**

Informational discontinuity can have far reaching consequences like medical errors, increased re-hospitalization rates and adverse events among others. Thus the holy grail of seamless informational continuity in healthcare has been an enigma with some nations going the digital way.

Digitization in healthcare in India is fast catching up. The current study explores the components of informational continuity, its impact on clinical decision-making and captures the general perception among the doctors towards a digital solution.

**Methods:**

Cross-sectional study with snowball sampling. A survey questionnaire was developed and validated through a pilot study, then circulated through online platforms. Responses from doctors were obtained through an online Google form for a period of 3 months and analyzed using SPSS 20. The categorical variables were analyzed using Chi-square test.

**Results:**

1413 responses were obtained through a national level survey. Respondents were from a wide range of work experiences, locations, sectors, specialties and patient load. Components of patient records like clinical notes, investigation reports, previous diagnosis and treatment details were rated to be very important.

41% reported about half and 20% reported about 3/4th of their patients do not bring relevant records. Patients from rural areas, visiting state government hospitals and visiting general practitioners were less likely to bring relevant records during consultations.

The fallouts of not having timely relevant patient information of the patients include more time per patient, repeat investigations, difficulty to arrive at definitive diagnosis, difficulty to take further treatment decisions and impaired overall clinical decision making which were said to be significant by respondents across the spectrum. The benefits of having timely relevant patient information were also reported consistently across the spectrum.

An overwhelming proportion (83%), from across the spectrum, unequivocally expressed their willingness to use digital platforms for accessing patients’ relevant medical records.

**Conclusion:**

Prevalence of informational discontinuity and its impact on clinical decision making is significant with definite benefits of having timely relevant medical history. There is strong willingness among the doctors to use digital solution(s) *without any extra investment or effort on their part* making customized solutions pertinent.

## Background

Information is a very important component of effective and efficient healthcare delivery. Exchange of information amongst the stakeholders is gaining importance especially with increasing complexity of healthcare. Free flow of information amongst patients, providers, insurance companies and government is important for smooth functioning of the healthcare system.

Information continuity is one among the important components of continuity of care along with management and relational continuity. Information is the common thread linking care from one provider to another and from one healthcare event to another [1]. Information can be disease or person focused. It is important in the process of patient care as it provides clarity on patient’s condition and events till date. This helps in clinical decision-making.

Discontinuity of care can have many deleterious effects like increased medical errors with higher rates of re-hospitalization [2]. There is unequivocal correlation between informational discontinuity and increase in adverse events [3]. Also adverse events have been found to decrease with interventions that ensure availability of timely, relevant information to treating teams [4].

Therefore ensuring informational continuity, which is a key component of continuity of care, has been a formidable challenge for healthcare systems across the world. Previous studies from the west had found that less than half of the PCPs (primary care physicians) got relevant information about their patients [5–7] which had cascading effects on overall patient care in terms of medical errors, higher readmissions among others.

In the quest for informational continuity many developed countries have over the period of time evolved digital platforms in the form of Electronic Health Record (EHR), Personal Health Record (PHR), health repositories etc. These are helping to link the various stakeholders in healthcare.

The United States of America has passed legislations like the Affordable Care Act 2010 and the Health Information Technology for Economic and Clinical Health Act (HITEC) 2009 which make it mandatory for healthcare providers to use EHR in order to get reimbursements.

However, the challenge of informational continuity remains to be enigmatic. The western countries have managed to achieve higher adoption of EHRs but at the cost of physician burnouts, lack of human touch with more screen time and interoperability issues. These can offer valuable lessons in terms of possible pitfalls that should be factored into the new upcoming systems.

The healthcare sector in India is poised for rapid, disruptive and unforeseen twists in view of changing demographics, better purchasing power, newer technologies and more demanding clientele. There is a growing drive towards digitization in Indian healthcare with the government aiming to provide digital health records to all its citizens by the year 2022.

The Ministry of Health & Family Welfare, Government of India has been taking initiatives and has put out Electronic Health Record guidelines in 2016 in this regard. The NITI Aayog (National Institution for Transforming India) has identified the importance of having a robust technology backbone and has come out with a proposal to establish “National Health Stack” (NHS). The NHS is envisioned to act as a platform to connect various stakeholders and support a multitude of health verticals and their disparate branches.

Digital solutions are the way to go but at the same time due regard needs to be given to the local conditions and constraints. In view of the fallouts seen in the western approach, merely mimicking the west for creating an information ecosystem for healthcare is not advisable. Instead we need to focus on developing customized solutions. The principle of having a robust information management system is apt but the approach we adopt in creating it will make all the difference.

Building customized solutions requires better understanding of the ground situation. Therefore to start with we need a better understanding on the informational continuity between the two most important stakeholders; the doctors and their patients. To the best of our knowledge *there are no scientific studies in this regard from India*.

Thus, the current study is aimed at obtaining more insights into the components of informational continuity, its impact on clinical decision-making and captures the general perception among the doctors regarding a possible digital solution to address this problem.

### Review of literature

Continuity is the degree to which a series of discrete healthcare events is experienced as coherent, connected and consistent with the patient’s medical needs and personal context. Three types of continuity exist in all settings: informational, management and relational. Continuity of care is achieved by bridging discrete elements in the care pathway - whether different episodes, interventions by different providers. For continuity to exist, care must be experienced as connected and coherent. The experience of continuity for providers is their perception that they have sufficient knowledge and information about a patient which helps them to best apply their professional competence. It also reflects as the confidence that their care inputs will be recognized and pursued by other providers [1].

A review article by Kriplani S et al. found that deficits in communication and information transfer at hospital discharge are common and may adversely affect patient care [8]. A study was carried out on General Practitioners (GPs) in England and the study reiterates the importance of informational continuity for better patient care and outcomes [9].

In the study by Small S et al. patient experience was seen from the perspective of informational discontinuity during care. It was found that the outcomes were linked to informational continuity, which was said to be the central component of effective healthcare delivery and patient management [10]. Delbanco T et al. in their study reiterated that information should travel with the patient (either physically or virtually). This would decrease information loss and potentially empower patients (and care providers) to access their information and serve as a source of continuity [11].

This brings us to discussions around computerization of health care. Here interoperability of computer systems is often presented as a pre-cursor for achieving continuity of care, as it is expected to support the coordination of information collected, stored, and shared with the aid of both human (e.g., care providers) and non-human (e.g., computer systems) systems, which together attempt to coordinate care across settings and over time [12].

### Aim

To study the impacts of gaps in informational continuity of care on clinical decision making among doctors and general perception about adoption of digital solutions.

### Objectives

1. To assess the perceived impact of informational discontinuity on clinical decision making by doctors.
2. To assess the general perception among doctors about adoption of digital solutions.

## Methods

### Type of study

Cross-sectional, descriptive study.

### Study period

January 2019 to March 2019.

### Sampling method

Snowball sampling through a national level survey.

### Study population

All M.B.B.S doctors who were willing to participate in the study.

A survey questionnaire was formulated by brainstorming followed by Focus Group Discussion among domain experts and clinicians. Some cues were taken from Massachusetts e-Health Collaborative (MAeHC) questionnaire for deciding on the structure of our questionnaire. This was done after obtaining due permission from the authors.

Brainstorming session was conducted with ten clinicians involved in general practice and broad specialties like Internal Medicine, Orthopaedics, Paediatrics, Obstetrics & Gynaecology, Psychiatry and Surgery. The session was moderated by a team of two hospital administrators and 2 independent reviewers who were clinicians.

Brainstorming was conducted based on the practical aspects and on-ground experiences of the participants with informational continuity of care. Then, broad areas that were found to be relevant to the participants were listed down by the moderators. Themes were ranked based on how frequently they appeared during the brainstorming session by the two independent reviewers.

These were then made into sections of the questionnaire like the profile of respondents, aspects/parts of patients’ records, proportion of patients not bringing relevant records in daily practice, fallouts of lack of relevant records, benefits of having relevant records and willingness among doctors to use digital solutions. Thus a broad structure of the questionnaire with different sections that covered various aspects was finalised from the brainstorming session.

Subsequently Focus Group Discussion (FGD) was held with the same group of clinicians, moderators and independent reviewers to further elaborate and add specific questions under each section. Sections of the questionnaire were deliberated upon. Recurring themes in each area/section were noted by the moderators. These themes were then ranked in their order of importance based on how strongly they impacted daily clinical practice. The participants (ten clinicians) decided on the ranking through voting.

This list of shortlisted themes was then separately reviewed and validated by the two independent reviewers who were also clinicians. Since the questionnaire had to be crisp to improve response rate and yet had to be comprehensive, it was decided to shortlist only the important and relevant themes through the above mentioned exercise. Themes thus identified were then made into questions and used in the questionnaire.

After obtaining permission from the institute ethics committee, validation was done through a Pilot study. In the pilot study, doctors were randomly selected from a list of doctors with varied qualifications, work profile and locations of practice from across the country and the questionnaire was sent to them through e-mail. Responses from the first 30 respondents was taken and analysed. The Cronbach’s alpha was found to be 0.754, indicating the internal consistency in the questionnaire. Thus the questionnaire was validated through the pilot study and then used for the current study. This questionnaire was then built on Google forms.

The total number of all doctors in India with their qualifications and geographical distribution was not available. Thus snowball sampling was used at national level to collect responses and was not restricted to any particular focus group.

The link for this Google form with the questionnaire was circulated through online platforms like WhatsApp, e-mail, Facebook and Curofy. It was circulated on all WhatsApp groups with only doctors, E-mails were sent to only doctors, Curofy and Facebook being social media platforms helped us to reach out to more doctors. The participants were also requested to further forward this study to doctors they know and so on.

Since the welcome statement mentioned this questionnaire was specifically meant for doctors and opening question clearly asked the participants their qualification, it is unlikely that people who are not doctors would have participated. Participants had to provide E-mail address or mobile number to answer the questionnaire. These were also used to prevent more than one response by a single participant.

Responses were obtained for period of 3 months from January 2019 to March 2019 after which no new entries were taken into the Google form with the questionnaire. The responses were analyzed using SPSS 20. The categorical variables were analyzed using Chi-Square test.

## Results

A total of 1413 responses were obtained for the questionnaire during the above mentioned duration. There were no missing values among these 1413 responses since there was no option to skip any question and final submission could not be done without answering all questions.

**Fig. 1:**
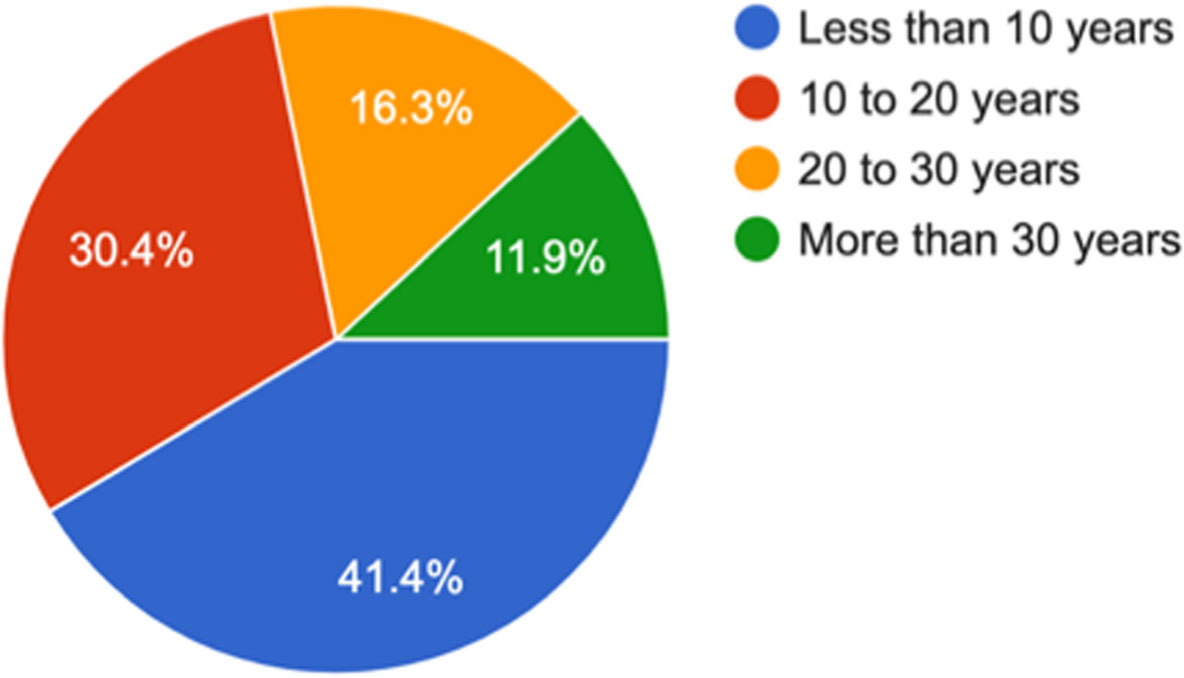
How many years of work experience post-MBBS? (1413 responses)

**Fig. 2:**
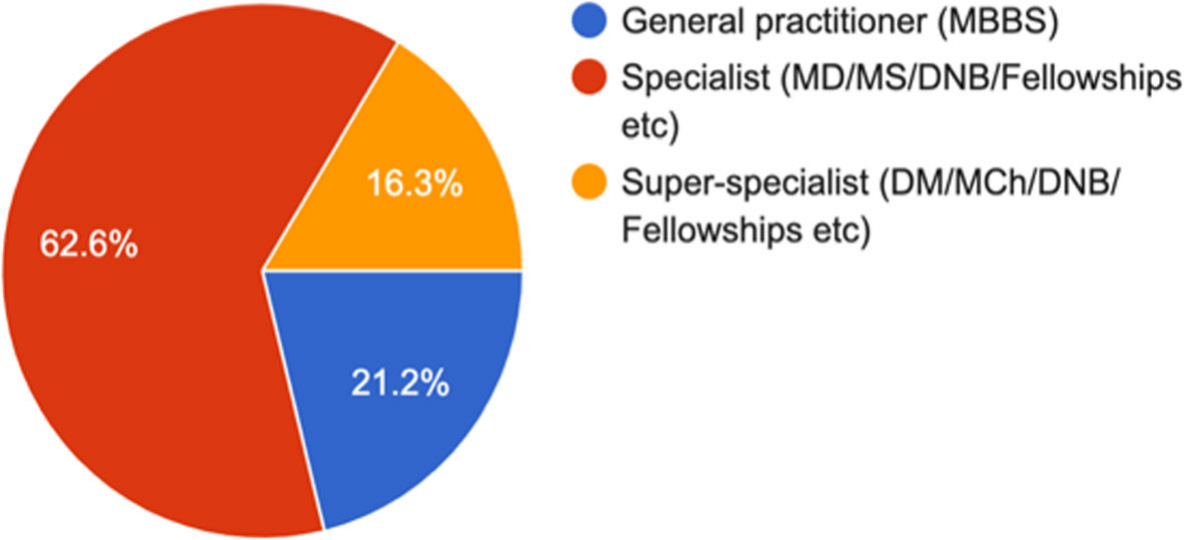
You are a? (1413 responses)

**Fig. 3:**
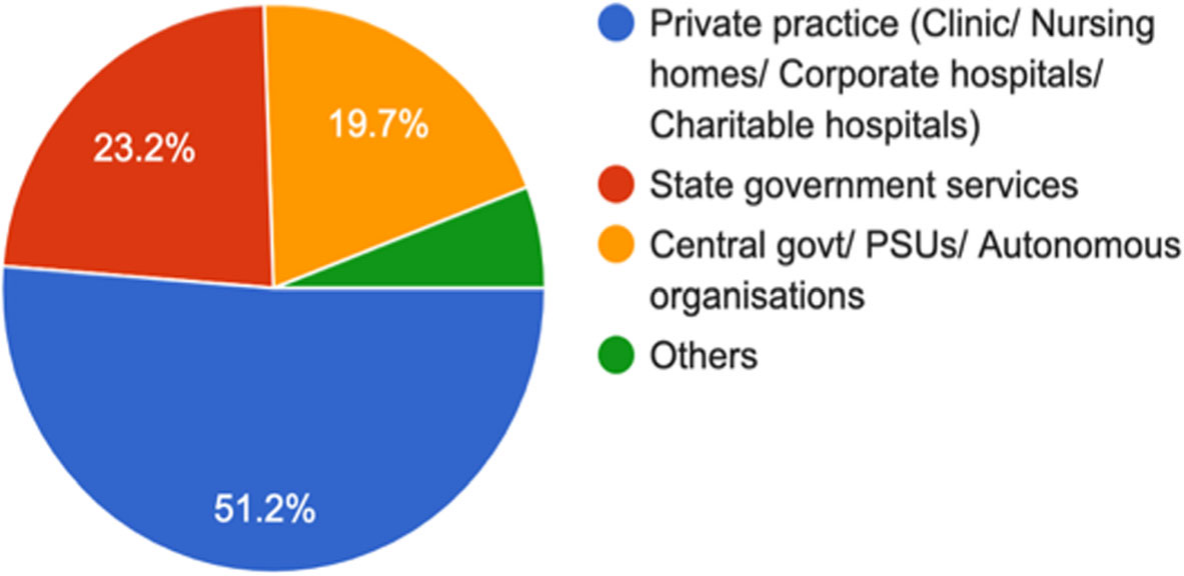
You work for? (1413 responses)

**Fig. 4:**
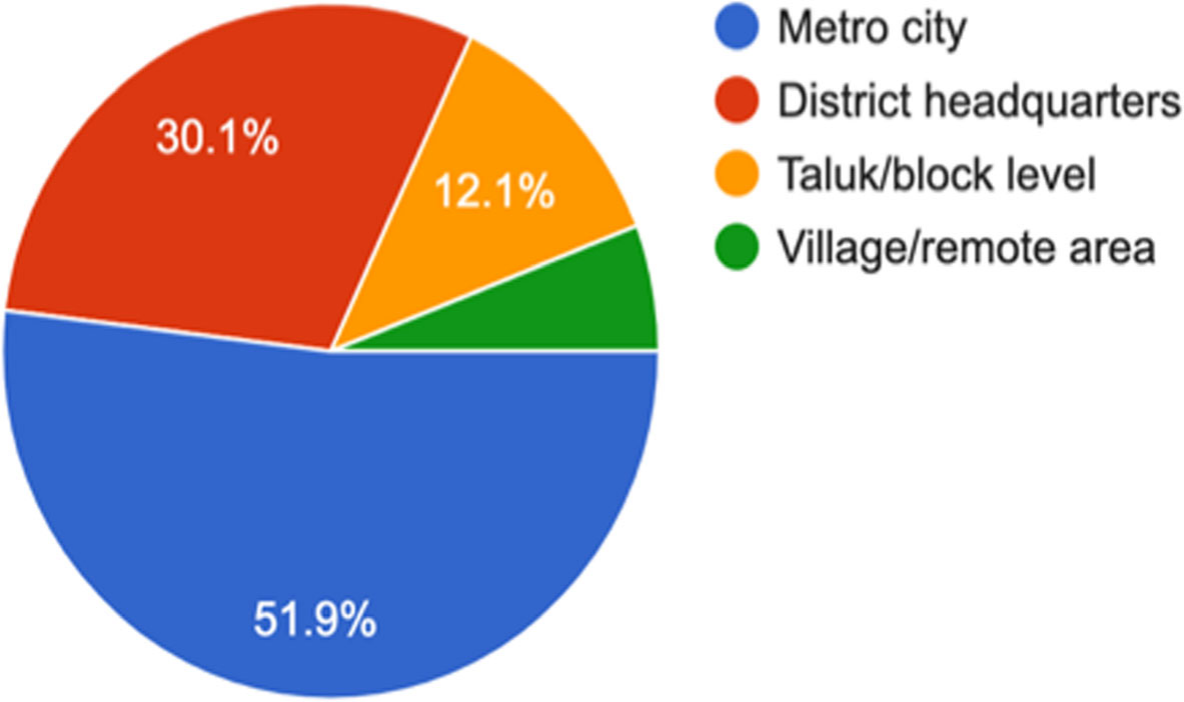
Area/location of practice? (1413 responses)

The following are the profile of respondents; years of experience, type of practitioner, sector, area/ location of practice and average number of patients seen per day. The following pie charts provide snapshots of each of the above-mentioned parameter. *(*Figs. 1*,*2*,*3*,*4*,* and 5*).*

**Fig. 5:**
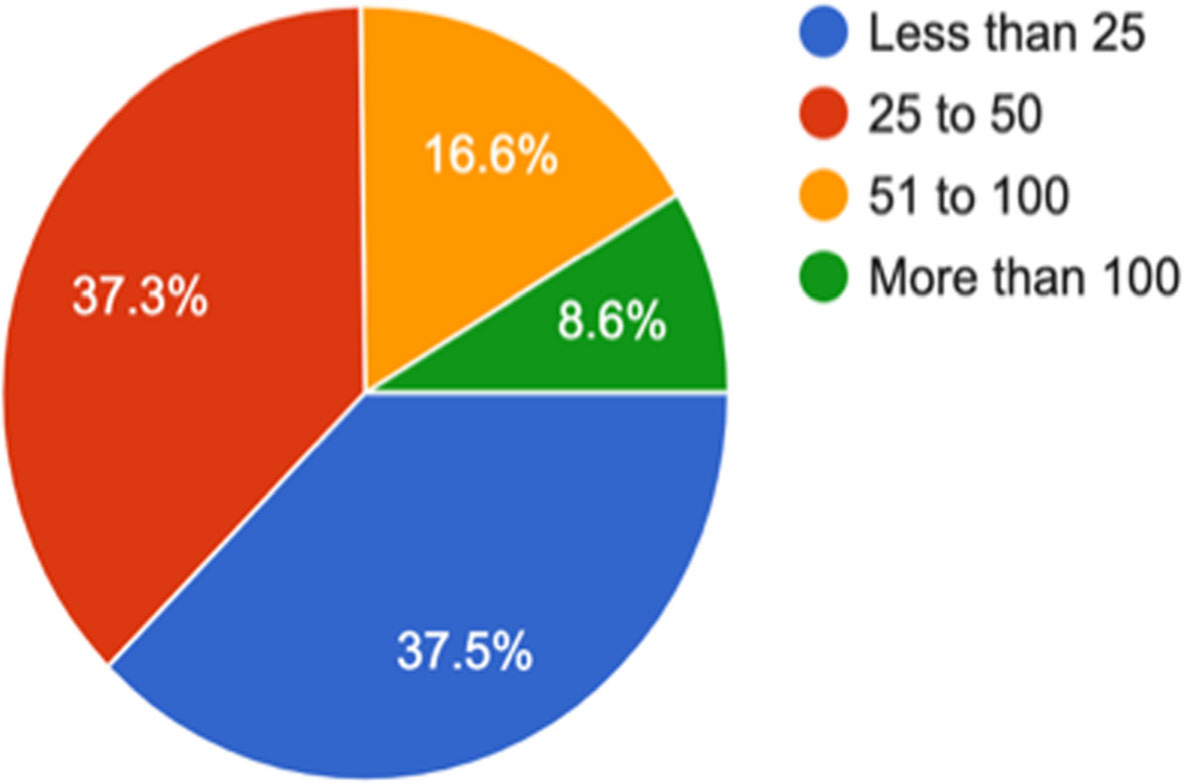
How many patients do you see daily on an average? (1413 responses)

There are various aspects/ parts of a patient’s records that provide the relevant medical history. The respondents have rated on a 5-pointlikert scale as to how important they perceive each aspect. *(*Table 1*).*

**Table 1:** Aspects/ parts of patients’ records

***n*** = 1413	Strongly disagree	Disagree	Neither agree nor disagree	Agree	Strongly Agree
Clinical notes	0.2%	1.1%	4.2%	17.8%	76.7%
Investigation reports	0.5%	1.6%	14.5%	33.6%	49.8%
Previous diagnosis	0.3%	2.5%	14.6%	29.6%	53%
Treatment details	0.4%	0.8%	9.1%	28.8%	60.9%
Immunization details	7.2%	10.5%	25.3%	26.8%	30.1%

**Fig. 6:**
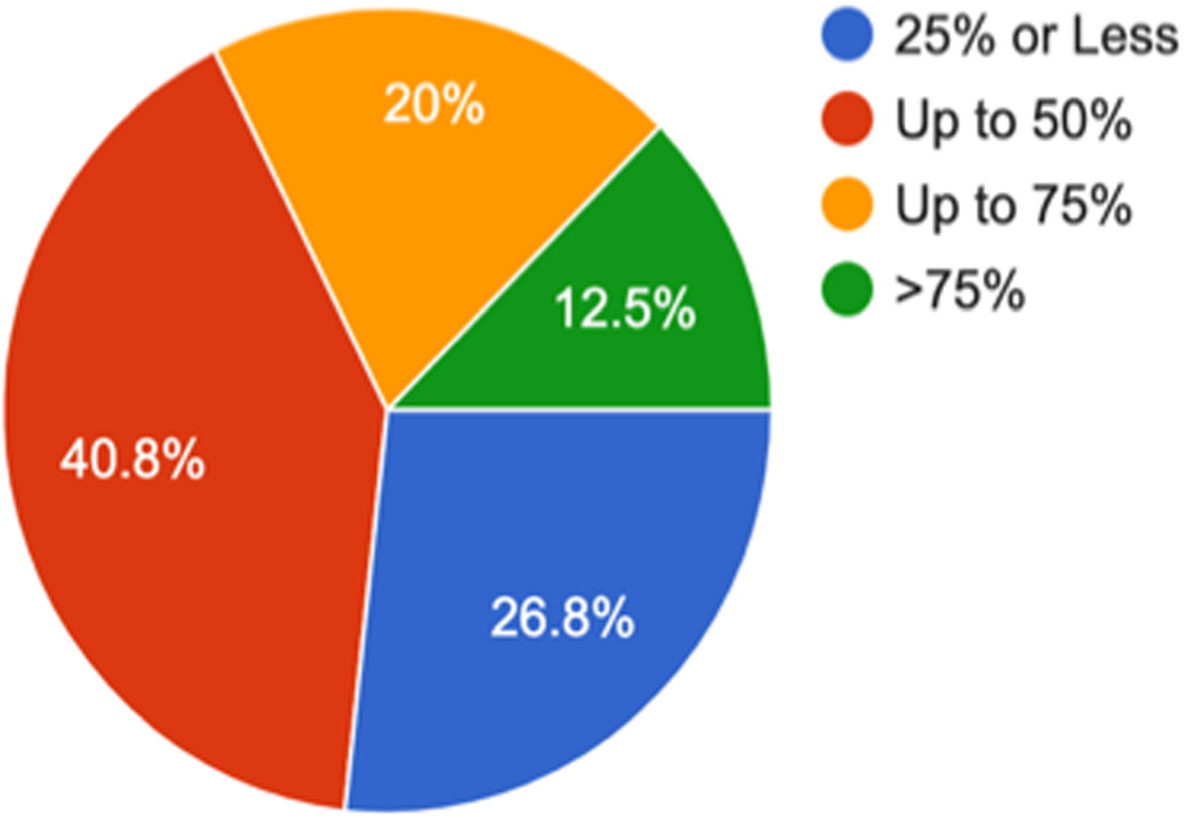
In your daily practice, around what % of your patients DO NOT bring relevant medical records/documents? (1413 responses)

The proportion of patients who do not bring relevant medical records is higher in rural areas and reported to be higher in state government organizations, which were found to be statistically significant as well. The proportion of patients who do not bring relevant medical records was reported to be higher by the General practitioners and lower by super-specialists, the difference between these two categories being statistically significant. This means that patients consulting super specialists are more likely to carry relevant medical records compared to patients going to general practitioner. (Fig. 6*).*

In the subsequent section of the questionnaire the respondents/ doctors rate the impacts of not having relevant medical records along a 5 point likert scale. The following table captures the perception of doctors in this regard: *(*Table 2*).*

**Table 2:** Impact/ fallouts of lack of relevant patient records

***n*** = 1413	Strongly disagree	Disagree	Neither agree nor disagree	Agree	Strongly Agree
Spend more time with patients	0.5%	1.1%	5.9%	26.3%	66.2%
Repeat investigations	1.3%	3.8%	18.9%	32.1%	43.9%
Difficult to arrive at definitive diagnosis	2.3%	9.1%	24.5%	34.9%	29.2%
Difficult to take further treatment decisions	2.1%	9.9%	23%	34.7%	30.3%
Impair overall clinical decision making	3.8%	9.6%	23.5%	32.2%	30.9%

The responses towards all options have been more towards agree and strongly agree. Across the spectrum, all different kinds of doctors/ respondents from all kinds of backgrounds & work experiences have consistently reported these challenges to be significant with no statistical difference among different kinds of respondents.

Timely availability of medical records/ information while seeing patients has its own innate advantages. The next section of the questionnaire gets respondents to rate the possible advantages of having timely relevant medical records along a 5 point likert scale. *(*Table 3*).*

**Table 3:** Advantages/ benefits of having relevant patient records

***n*** = 1413	Strongly disagree	Disagree	Neither agree nor disagree	Agree	Strongly Agree
It will save time	0.6%	0.8%	5%	18.2%	75.3%
Easy to arrive at a definitive diagnosis	0.5%	1.6%	8.6%	27.5%	61.8%
Easy to take treatment decisions	0.6%	1.1%	6.9%	29.8%	61.6%
Decrease overall burden/ load	1.2%	2.1%	9.6%	25.3%	61.9%

The responses for all options have been more towards agree and strongly agree. Respondents from across the board from different backgrounds, work experiences, sectors and locations have agreed or strongly agreed that these are the advantages if relevant medical records are made available.

After capturing the perception of the respondents in terms of problems posed due to lack of relevant medical records and benefits of having timely records, we need to look into possible solutions. Digitization being one of the important solutions, it is pertinent to know the level of willingness among doctors to use any form of digital tool/ solution. In the Indian context, the adoption of EHR systems is very low and unlikely to increase as there is no legal or policy compulsion to use them. Besides these systems are generally expensive making them even less attractive for doctors.

Therefore any plausible digital solution ought to be without any extra effort or investment on part of the doctors. Extra efforts in terms of learning and manual entry of data by doctors deter adoption of digital solutions. Intuitive applications that can capture data with minimal manual data entries are required.

**Fig. 7:**
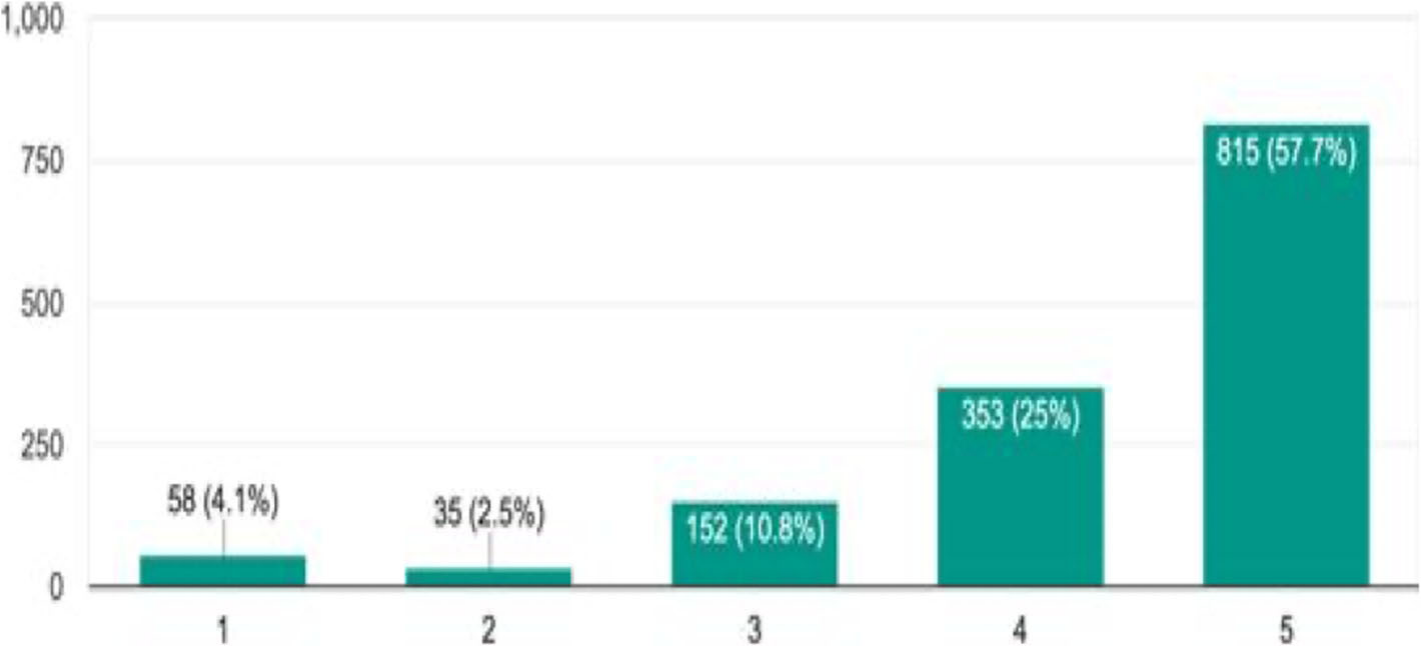
Without any extra investment or effort from your side, if relevant medical documents/information are made available to you through your smartphone/tablet/ computer, will you use it?. (1-Strongly disagree, 2-Disagree, 3-Neither agree nor disagree, 4-Agree & 5-. Strongly Agree) (*n* = 1413)

Innovative revenue models that do not levy user charges and ease of deployment on existing or commonly available devices like smartphones, desktops among others would be highly desirable. These aspects have thus been reflected in the following question. *(*Fig. 7*).*

An overwhelming proportion said they strongly agree (57.7%) and agree (25%) that they are willing to use digital platforms for accessing patients’ relevant medical records. Respondents from across the board expressed their willingness without any statistical difference in the responses between the doctors with different work experiences, locations, sectors, profile or patient load.

The limitation of the study however is that the whole survey was conducted only through online platforms like e-mails, WhatsApp, Facebook and Curofy. Therefore the doctors who do not use these platforms could have been missed. Besides the response rate could not be captured as snowball sampling was used. This could result in only doctors who are interested and willing to discuss this topic being included, whereas it is difficult to know if the more resistant perspectives chose not to respond.

## Discussion

The western countries have been on the quest for the holy grail of informational continuity through early adoption of EHR systems. Legislative backing &market forces among other factors have facilitated wider adoption of EHR systems in these countries. In the Unites States especially, the “carrot and stick” approach of the HITECH (Health Information Technology for Economic and Clinical Health) Act and the ACA (Affordable Care Act) have dramatically increased the adoption of EHRs, which has in turn increased the availability of electronic health data. If not for these, most of the healthcare data would have been in the paper format [13]. However, it has come with its own set of challenges.

First, many studies have emphasized on the clinicians burnouts. Clerical burden including documentation of care and order entry are the major drivers for clinician burnout [14] which take away about 50% of their time. Even nurses spend about half their time fulfilling documentation requirements [15]. These have also decreased the time doctors spend making eye contact and doing physical examination [16] showing lack of human touch and empathy. Some studies have claimed that computer screens create a physical and psychological barrier between the clinicians and their patients [17].

Second, there have also been issues with disruption of clinical workflows/processes. Information systems embody the understanding of the developers/creators about the domain where they are implemented. It is important for the developers/creators to have a thorough understanding of how the work is done, characteristics of the environment and the people who work there and how they will use the system. More often than not, the basis/rules used for development of these computer systems are different from the actual processes of clinical work [18]. The end result being, these information systems are often forced down upon the clinicians whose way of working is quite different. In essence *“The processes and systems are forced to fit into the software rather than other way round.”*

Wears RL et al. have rightly remarked in their research work that [18]-

*“Clinical work, especially in hospitals, is fundamentally interpretative, interruptive, multitasking, collaborative, distributed, opportunistic, and reactive... The result of this mismatch is that many of the failed attempts at computer-based clinical systems were bound to fail because the model of health care work inscribed in these tools clashed too much with the actual nature of clinical work.”* [18]

**Fig. 8:**
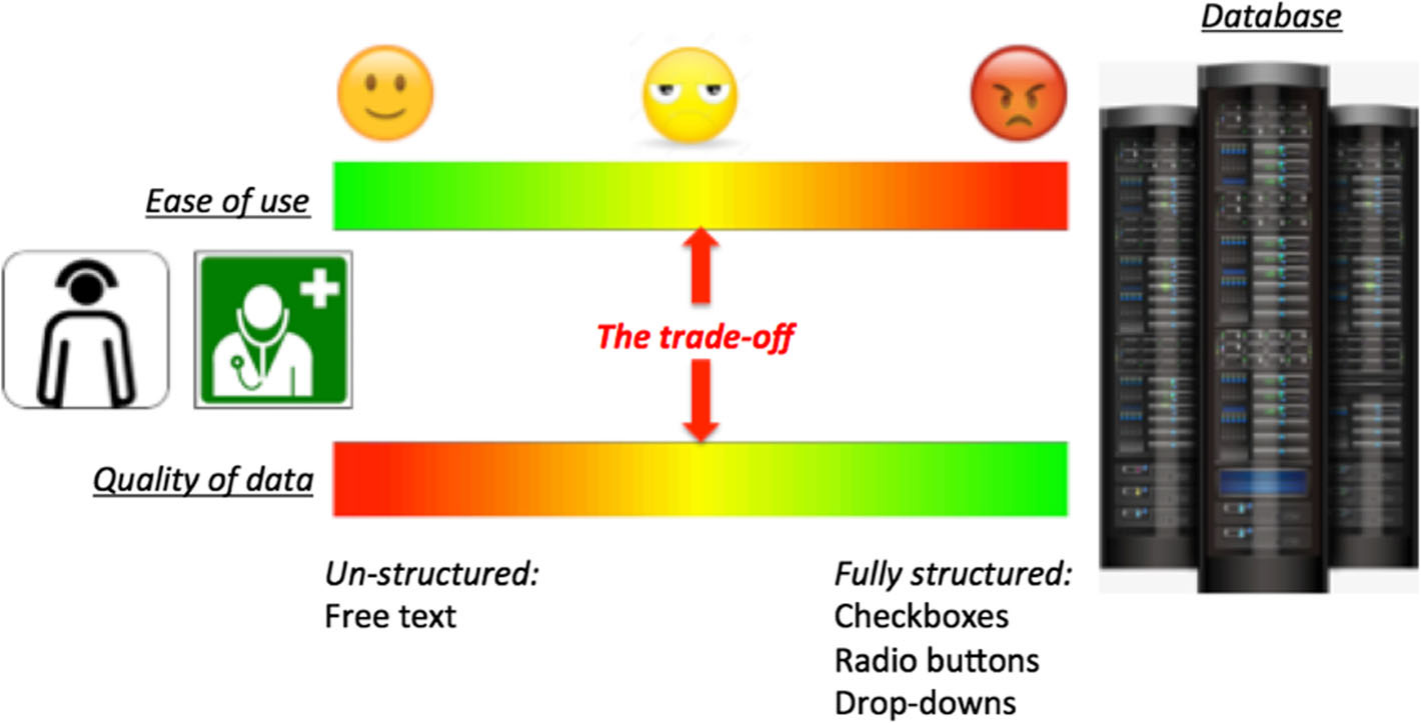
Conventional HIS/EHR: An expensive trade off

Third, the lack of user friendliness is increasingly being voiced which in turn affects compliance and burnouts among healthcare providers. In a quest towards collecting more structured data, there has always been a trade-off between having checkboxes/ radio-buttons and free text fields [13]. More the checkboxes, radio-buttons or drop-downs, more will be the structured data. But the fall-out is that in the process of forcefully categorizing into one of the drop-downs or radio-buttons, the actual narrative of the patients’ story is lost [19]. (Fig. 8)*.*

The following diagram illustrates the limitations with the conventional HIS/EHR systems. In order to get more structured data the system has to be made more rigid in the form of checkboxes, radio buttons among others which decreases the ease of use. If free text boxes with less rigid interfaces are provided to improve ease of use, unstructured data is generated, in turn compromising on the quality of the data. Therefore posing an expensive trade-off by providing either ease of use or quality data.

Information processing theory states that any increase in burden of entering data will have detrimental effects on data consistency and data quality [13]. Every increase in the clicks required either brings down compliance or can culminate in increased workload and burnouts.

Currently in India, the penetration of Electronic Health Records (EHR) is dismally low, though no formal figures are available. A few corporate hospitals have their own EHR systems with little or no interoperability [20]. This is an opportunity as healthcare IT in India is largely Greenfield. We would benefit the most if we learn from the pitfalls and mistakes of the west and leapfrog the development process.

Digitization of healthcare in India is still in its infancy. The proposed National Health Stack (NHS) and the subsequent National Digital Health Blueprint are steps in the right direction. However, any tendencies towards centralization and over regulation could stifle innovations.

Healthcare analytics broadly has stage of data capture/ extraction, stage of data analytics and that of data visualization [13]. A centralized data repository with open APIs (Application Program Interfaces) gives more room for innovative tools for stage of data analytics and visualization.

A decentralized, distributed system with scope for end users to adopt/create customized applications provides more scope for innovations in data capture/extraction, which is the need of the hour in Indian setting. There is tremendous scope to leverage AI (Artificial Intelligence) tools as advanced workflow technologies to suite Indian conditions. This would help to make healthcare digitization market driven rather than a top down enforcement.

## Conclusion

Through this study it is clear that prevalence of informational discontinuity and its impact on clinical decision making is significant as perceived by doctors. The benefits of having timely relevant medical history are also unequivocally established. There is strong willingness among the doctors to use digital solution(s) without any extra investment or effort on their part.

Any intuitive digital solution that requires minimum effort in form of manual data entry, does not levy user charges and can run on commonly used devices could be a game-changer. The ground is thus clear for e-health initiatives, but the success of any such initiative would depend on the policies and technologies that are leveraged.

## Supplementary information

**Additional file 1. **Questionnaire. Survey tool used in the study.

## Data Availability

The datasets used and/or analysed during the current study are available from the corresponding author on reasonable request.

## Abbreviations

ACA: Affordable Care Act
AI: Artificial Intelligence
AIIMS: All India Institute of Medical Sciences
APIs: Application Program Interfaces
EHR: Electronic Health Record
GPs: General Practitioners
HIS: Hospital Information system
HITEC: Health Information Technology for Economic and Clinical Health Act
IT: Information technology
MAeHC: Massachusetts eHealth Collaborative
NHS: National Health Stack
NITI Aayog: National Institution for Transforming India Aayog
PCPs: Primary care physicians
PHR: Personal Health Record
