# Supplementary material for: The information imperative: to study the impact of informational discontinuity on clinical decision making among doctors
Source: BMC Med Inform Decis Mak. 2020 Jul 28;20:175. doi: 10.1186/s12911-020-01190-2 (PMC7388506; doi:10.1186/s12911-020-01190-2)
Supplement: Supplementary file 1 — Additional file 1. Questionnaire. Survey tool used in the study. [file 12911_2020_1190_MOESM1_ESM.docx]

**ANNEXURE II**

**(Questionnaire)**

1. E-mail address: …………………………………………..
2. Mobile No: ………………………………………………….
3. How many years of work experience post-MBBS?
4. Less than 10 years
5. 10 to 20 years
6. 20 to 30 years
7. More than 30 years
8. You are a:
9. General practitioner (MBBS)
10. Specialist (MD/MS/DNB/Fellowships etc)
11. Super-specialist (DM/MCh/DNB/Fellowships etc)
12. Your specialty/ Sub-specialty: ……………………………….
13. You work for:
14. Private practice (Clinic/ Nursing homes/ Corporate hospitals/ Charitable hospitals)
15. State government services
16. Central govt/ PSUs/ Autonomous organizations
17. Others: …………………………………….
18. Area/location of practice:
19. Metro city
20. District headquarters
21. Taluk/block level
22. Village/remote area
23. How many patients do you see daily on an average?
24. Less than 25
25. 25 to 50
26. 51 to 100
27. More than 100
28. How important are the following aspects/information regarding your patients, in your clinical decision making and management? Rate them 1 to 5*

(1-least important, 2- somewhat important, 3- Important, 4- Very important & 5- extremely important)

| Clinical notes (symptoms and examination findings) | 1 | 2 | 3 | 4 | 5 |
| --- | --- | --- | --- | --- | --- |
| Investigation reports | 1 | 2 | 3 | 4 | 5 |
| Details of previous diagnoses | 1 | 2 | 3 | 4 | 5 |
| Treatment details | 1 | 2 | 3 | 4 | 5 |
| Immunization details | 1 | 2 | 3 | 4 | 5 |

1. In your daily practice around, what percentage of your patients bring relevant medical records/documents:

a. 25% or Less

b. Up to 50%

c. Up to 75%

d. >75%

1. When your patients do not bring relevant medical records/ documents:

|  | Strongly Agree | Agree | Neither agree nor disagree | Disagree | Strongly disagree |
| --- | --- | --- | --- | --- | --- |
| You have to spend more time with patients to get relevant information |  |  |  |  |  |
| You have to repeat investigations |  |  |  |  |  |
| It is difficult to arrive at definitive diagnosis |  |  |  |  |  |
| It becomes difficult to take further treatment decisions |  |  |  |  |  |
| It impairs your overall clinical decision making |  |  |  |  |  |
| Any other problems that you face |  | | | | |

1. If all the relevant medical records/ information are made available to you when seeing your patients:

|  | Strongly Agree | Agree | Neither agree nor disagree | Disagree | Strongly disagree |
| --- | --- | --- | --- | --- | --- |
| It will save my time |  |  |  |  |  |
| It is easy to arrive at a definitive diagnosis |  |  |  |  |  |
| It is easy to take treatment decisions |  |  |  |  |  |
| It will decrease my overall load/ burden |  |  |  |  |  |
| Any other benefits that you feel |  | | | | |

1. How often do you use the Internet for personal and/or professional purposes?
2. Several times a day
3. Daily
4. Weekly
5. Monthly
6. Less than monthly or not at all
7. Without any extra investment or effort from your side, if relevant medical documents/information are made available to you through your smartphone/ tablet/ computer, will you use it?

| Strongly Agree | Agree | Neither agree nor disagree | Disagree | Strongly disagree |
| --- | --- | --- | --- | --- |

1. Suggestions:

________________________________________________________________________________________________________________________________________________________________________________________________________________________________________________________________________________________________
